# Referral Rates for Cardiac Rehabilitation Among Eligible Inpatients After Implementation of a Default Opt-Out Decision Pathway in the Electronic Medical Record

**DOI:** 10.1001/jamanetworkopen.2020.33472

**Published:** 2021-01-14

**Authors:** Srinath Adusumalli, Elizabeth Jolly, Neel P. Chokshi, Yevginiy Gitelman, Charles A. L. Rareshide, Daniel M. Kolansky, Mitesh S. Patel

**Affiliations:** 1 Division of Cardiovascular Medicine, Perelman School of Medicine, University of Pennsylvania, Philadelphia; 2 Division of General Internal Medicine, Perelman School of Medicine, University of Pennsylvania, Philadelphia; 3 Office of the Chief Medical Information Officer, University of Pennsylvania, Philadelphia; 4 Penn Medicine Nudge Unit, Penn Medicine Center for Healthcare Innovation, Philadelphia, Pennsylvania; 5 The Wharton School, University of Pennsylvania, Philadelphia; 6 Corporal Michael J. Crescenz VA Medical Center, Philadelphia, Pennsylvania

## Abstract

This quality improvement study assesses referral rates for cardiac rehabilitation after a default opt-out option is added to the decision pathway in the electronic medical record.

## Introduction

Ischemic heart disease is the leading cause of mortality in the United States.^1^ Cardiac rehabilitation (CR) is an evidence-based therapy that reduces mortality, morbidity, and hospital readmissions in patients with ischemic heart disease.^2^ However, CR is widely underused: 25% of US hospitals refer less than 20% of eligible patients.^3^ Novel scalable approaches are needed to improve CR referral rates.^4^

Default options are the path of least resistance and set conditions that occur if no alternative is chosen.^5^ Previous work has shown how changing default settings can significantly influence clinicians’ prescribing behaviors.^6^ Default options may also influence more complex decision pathways, but this tactic has not been well examined.

In this quality improvement study, we evaluated changes in CR referral after automated electronic health record–based technology was used to identify eligible patients and decision pathways were redesigned from opt-in to opt-out referral. We compared changes that occurred across 3 years at an intervention hospital with changes in 2 control hospitals in the same academic health system.

## Methods

The study period extended from January 1, 2016, to December 31, 2018. From September to December 2016, we experimented with redesigning defaults in the decision pathway at 1 of 3 Penn Medicine hospitals in Philadelphia, Pennsylvania. In January 2017, we implemented an opt-out CR referral decision pathway that used the electronic health record to automatically identify eligible patients from the electronic health record and notify appropriate staff on the wards by using secure text messaging (eMethods in the Supplement). Rounds were restructured so that cardiologists signed templated CR orders and staff met with patients to facilitate CR placement before discharge. Appropriate CR referrals were manually verified. We also created educational material for the patient on the importance and relevance of CR therapy, which was provided to the patient by the clinical resource coordinator. This study was reviewed and determined to qualify as a quality improvement study by the University of Pennsylvania institutional review board. Patient informed consent was waived because the study was primarily a quality improvement and operation project advancing guideline-directed standard of care. This study followed the Standards for Quality Improvement Reporting Excellence (SQUIRE) guideline.

Data for 1 year before and 2 years after the intervention were obtained from medical record–abstracted CR referral rates submitted to the CathPCI Registry, one of the registries in the National Cardiovascular Data Registry administered by the American College of Cardiology.^4^ A linear probability model was used to perform a difference-in-differences analysis to evaluate changes in CR referral rates at the intervention site vs the 2 control sites during a 1-year preintervention period and a 2-year postintervention period, excluding the washout period of rapid experimentation. The unit of analysis was the patient, and the model included variables for time (before vs after the intervention), site (intervention vs controls), and an interaction term for time and site. A test of control sites was conducted in the first 9 months of year 1 to evaluate pre-intervention trends. All models were adjusted for age, sex, race/ethnicity (obtained from the electronic heart record based on patient report), insurance, annual household income, body mass index, and history of myocardial infarction, congestive heart disease, cerebrovascular disease, chronic obstructive pulmonary disease, hypertension, diabetes, and smoking. Two-sided hypothesis tests used a significance level of .05; SAS statistical software, version 9.4 (SAS Institute Inc) was used for all analyses.

## Results

The sample consisted of 2832 patients with ischemic heart disease at 3 hospital sites. The patients had a mean (SD) age of 66.7 (11.4) years; 812 (28.7%) were female, 1870 (66.0%) 66.0% were White, and 564 (19.9%) were Black (Table). The Figure shows CR referral rates by site over time. Preintervention trends did not differ between the intervention and control sites. Preintervention trends did not differ between the intervention and control sites. At the end of the study, the percentage of CR referrals at the intervention site was 85.7% and for the control sites was 31.6%. Compared with the control sites over time, the intervention site had a significant 47–percentage point increase in CR referrals (95% CI, 39.2-55.1 percentage points; *P* < .001).

**Table.** Sample Characteristics

Characteristic	Site by year of study, No. (%)						
	2016		2017		2018		Total, all years (N = 2832)
	Control (n = 662)	Intervention (n = 258)	Control (n = 778)	Intervention (n = 242)	Control (n = 698)	Intervention (n = 194)	
Age, mean (SD), y	67 (11.8)	66.1 (12.5)	67.3 (10.8)	64.2 (11.4)	67.0 (10.9)	65.8 (12.4)	66.7 (11.4)
Female	206 (31.1)	70 (27.1)	233 (29.9)	56 (23.1)	196 (28.1)	51 (26.3)	812 (28.7)
Race/ethnicity							
Non-Hispanic White	451 (68.1)	156 (60.5)	523 (67.2)	156 (64.5)	476 (68.2)	108 (55.7)	1870 (66.0)
Non-Hispanic Black	133 (20.1)	59 (22.9)	155 (19.9)	51 (21.1)	123 (17.6)	43 (22.2)	564 (19.9)
Other^a^	78 (11.8)	43 (16.7)	100 (12.9)	35 (14.5)	99 (14.2)	43 (22.2)	398 (14.1)
Insurance							
Commercial	241 (36.4)	110 (42.6)	265 (34.1)	115 (47.5)	249 (35.7)	77 (39.7)	1057 (37.3)
Medicare	369 (55.7)	130 (50.4)	459 (59.0)	112 (46.3)	394 (56.4)	97 (50.0)	1561 (55.1)
Medicaid	52 (7.9)	18 (7.0)	54 (6.9)	15 (6.2)	55 (7.9)	20 (10.3)	214 (7.6)
Annual household income, $							
<50 000	260 (39.3)	98 (38.0)	310 (39.8)	79 (32.6)	262 (37.5)	72 (37.1)	1081 (38.2)
50 000-100 000	361 (54.5)	128 (49.6)	406 (52.2)	114 (47.1)	379 (54.3)	86 (44.3)	1474 (52.0)
>100 000	34 (5.1)	29 (11.2)	49 (6.3)	46 (19.0)	49 (7.0)	32 (16.5)	239 (8.4)
Missing	7 (1.1)	3 (1.2)	13 (1.7)	3 (1.2)	8 (1.1)	4 (2.1)	38 (1.3)
Myocardial infarction	345 (52.1)	80 (31.0)	421 (54.1)	115 (47.5)	380 (54.4)	107 (55.2)	1448 (51.1)
Congestive heart failure	193 (29.2)	52 (20.2)	271 (34.8)	61 (25.2)	220 (31.5)	78 (40.2)	875 (30.9)
Cerebrovascular disease	92 (13.9)	30 (11.6)	126 (16.2)	44 (18.2)	123 (17.6)	34 (17.5)	449 (15.9)
COPD	155 (23.4)	39 (15.1)	183 (23.5)	51 (21.1)	153 (21.9)	46 (23.7)	627 (22.1)
Hypertension	495 (74.8)	127 (49.2)	639 (82.1)	170 (70.2)	575 (82.4)	133 (68.6)	2139 (75.5)
Diabetes	264 (39.9)	73 (28.3)	343 (44.1)	85 (35.1)	285 (40.8)	89 (45.9)	1139 (40.2)
Smoking history							
Current	63 (9.5)	16 (6.2)	129 (16.6)	27 (11.2)	114 (16.3)	21 (10.8)	370 (13.1)
Ex-smoker	224 (33.8)	82 (31.8)	333 (42.8)	95 (39.3)	297 (42.6)	80 (41.2)	1111 (39.2)
Never	153 (23.1)	75 (29.1)	251 (32.3)	92 (38.0)	243 (34.8)	65 (33.5)	879 (31.0)
Missing	222 (33.5)	85 (32.9)	65 (8.4)	28 (11.6)	44 (6.3)	28 (14.4)	472 (16.7)
BMI, mean (SD)	30.1 (6.2)	29.5 (6.9)	30.3 (6.2)	29.4 (5.4)	29.9 (5.9)	29.3 (5.6)	29.9 (6.1)
Missing, No.	21	21	12	5	7	0	66
Charlson Comorbidity Index, median (IQR)	2 (1-4)	1 (0-4)	2 (1-5)	2 (1-4)	2 (1-4)	3 (1-5)	2 (1-4)

Abbreviations: BMI, body mass index (calculated as weight in kilograms divided by height in meters squared); COPD, chronic obstructive pulmonary disease; IQR, interquartile range.

^a^ Other race/ethnicity is an independent category in the electronic health record that can be selected by the patient.

**Figure.**
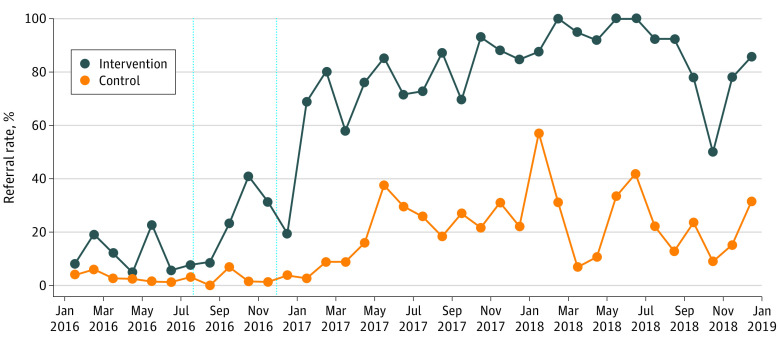
Cardiac Rehabilitation Referral Rates by Site and Time

## Discussion

The findings of this quality improvement study suggest that default options can be used for more complex decision pathways because the intervention was associated with a sustained significant increase in CR referrals. This increase may be due to a shift in effort on the part of cardiologists who previously had to manually opt-in to refer patients and now were prompted to sign orders during rounds unless they opted-out. This method facilitated CR referral by optimizing cardiologists’ workflow and sharing tasks with other staff.

At the control sites, CR referrals increased in the second quarter of 2017, which may have been due to cardiologists becoming aware of initial results at the intervention site. However, this increase was not as substantial as that at the intervention site and highlights the importance of combining education with behavioral change strategies. This study was limited by its observational design at only 1 health system and by the lack of follow-up data on patient CR participation rates. In conclusion, restructuring decision pathways from an opt-in to an opt-out choice was associated with a significant increase in CR referrals. This pathway represents a low-cost, scalable approach that could be expanded to other health systems and for other therapies.

## Notes

Supplement. **eMethods.** Decision Pathway **eReference.**
