# Supplementary material for: Referral Rates for Cardiac Rehabilitation Among Eligible Inpatients After Implementation of a Default Opt-Out Decision Pathway in the Electronic Medical Record
Source: JAMA Netw Open. 2021 Jan 14;4(1):e2033472. doi: 10.1001/jamanetworkopen.2020.33472 (PMC7809585; doi:10.1001/jamanetworkopen.2020.33472)
Supplement: Supplement. — eMethods. Decision Pathway eReference. [file jamanetwopen-e2033472-s001.pdf]

SUPPLEMENTARY ONLINE CONTENT

Adusumalli S, Jolly E, Chokshi NP, et al. Referral rates for cardiac rehabilitation among eligible inpatients after implementation of a default opt-out decision pathway in the electronic medical record. *JAMA Netw Open.* 2021;4(1):e2033472. doi.10.1001/jamanetworkopen.2020.33472

eMethods. Decision Pathway

eReference.

This supplementary material has been provided by the authors to give readers additional information about their work.

© 2021 Adusumalli S et al. *JAMA Network Open.*

eMethods. Decision Pathway.

We implemented an electronic health record (EHR)-based opt-out decision pathway to increase cardiac rehabilitation (CR) referrals among eligible patients with ischemic heart disease (IHD) presenting with stable ischemic heart disease or acute coronary syndrome. The decision pathway leveraged the EHR to identify eligible patients based on diagnosis of acute myocardial infarction as well as relevant procedures (percutaneous coronary intervention).

The decision pathway consisted of the following steps:

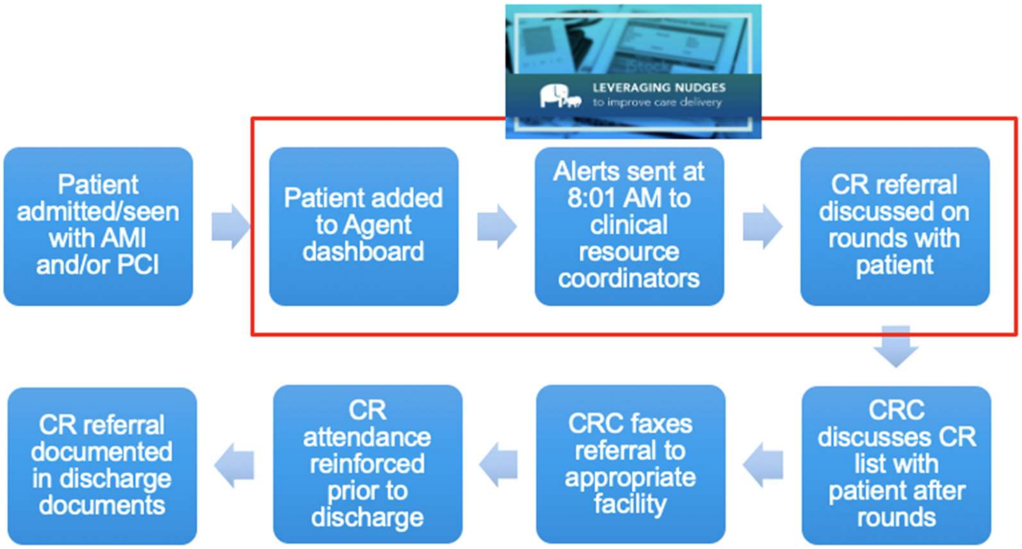

*CRC stands for clinical resource/transition coordinator. Within our health system, CRCs coordinate post-discharge care. As part of our intervention, CR was added to the list of options/recommendations for post-discharge care discussed by the CRC with patients (similar to a skilled nursing facility). In addition to the steps in this pathway, a task to consider CR referral was also added to provider handoff tools as a form of non-interruptive clinical decision support. We additional created a fully templated paper order for CRCs to bring to providers to sign (providers could opt-out of referral) and a database of CR facilities by location to make it easy to discuss referral with patients. Finally, we created educational material for the patient on the importance and relevance of CR therapy that the CRC provided to the patient.

© 2021 Adusumalli S et al. *JAMA Network Open.*

Example of secure text message and screenshot from Agent platform which sent secure text messages and through which CRCs managed referrals:

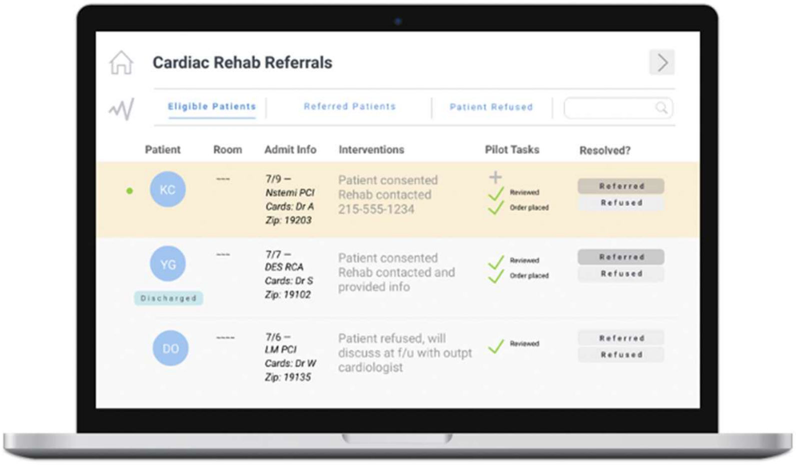

Figure courtesy of Katherine Choi, MD

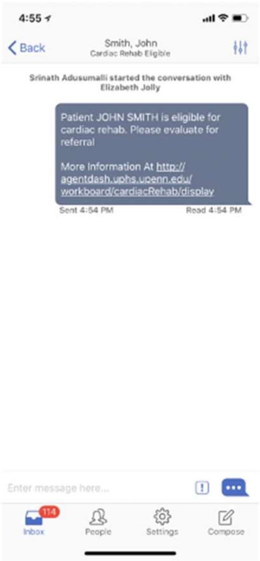

To learn more about the Agent platform, see Choi et al.[1]

This intervention could also be implemented without the Agent platform and within the context of common electronic health record platforms by determining eligibility through the presence of relevant AMI diagnosis codes or PCI procedure codes in association with prescription of relevant medications such as aspirin and P2Y12 antagonists. Discrete documentation tools which generate data elements could also be used to trigger decision support and the CR referral process. Options for automated decision support to providers and staff assisting with referral within the EHR include interruptive alerts, noninterruptive work queues/pools monitored by ancillary/support staff, messaging, and use of tasks placed within handoff tools.

eReference

1. Choi K, Gitelman Y, Asch DA. subscribing to your patients—reimagining the future of electronic health records. *New England Journal of Medicine.* 2018;378(21):1960-1962. doi:10.1056/NEJMp1800874

© 2021 Adusumalli S et al. *JAMA Network Open.*
